# Patterns in Psychiatrists’ Prescription of Valproate for Female Patients of Childbearing Age With Bipolar Disorder in Japan: A Questionnaire Survey

**DOI:** 10.3389/fpsyt.2020.00250

**Published:** 2020-04-15

**Authors:** Masumi Tachibana, Tasuku Hashimoto, Mami Tanaka, Hiroyuki Watanabe, Yasunori Sato, Takashi Takeuchi, Takeshi Terao, Shou Kimura, Akio Koyama, Sachie Ebisawa, Yuichiro Shizu, Teruyoshi Nagase, Junichi Hirakawa, Kotaro Hatta, Michiko Nakazato, Masaomi Iyo

**Affiliations:** ^1^ Department of Psychiatry, Chiba University Graduate School of Medicine, Chiba, Japan; ^2^ Department of Psychiatry, Sodegaura-Satsukidai Hospital, Sodegaura, Japan; ^3^ Division of Clinical Study on Juvenile Delinquency, Center for Forensic Mental Health, Chiba University, Chiba, Japan; ^4^ Division of Medical Treatment and Rehabilitation, Chiba University Center for Forensic Mental Health, Chiba, Japan; ^5^ Department of Psychiatry, Gakuji-kai Kimura Hospital, Chiba, Japan; ^6^ Department of Preventive Medicine and Public Health, School of Medicine, Keio University, Tokyo, Japan; ^7^ Department of Psychosomatic and Palliative Medicine, Tokyo Medical and Dental University Medical Hospital, Bunkyo-ku, Japan; ^8^ Department of Neuropsychiatry, Faculty of Medicine, Oita University Yufu, Oita, Japan; ^9^ Department of Psychiatry, Yurakucho Sakura Clinic, Chiyoda-ku, Japan; ^10^ Department of Psychiatry, Alba Mental Clinic, Shinjuku, Japan; ^11^ Shizu Clinic, Sakura, Japan; ^12^ Department of Psychiatry, Takatsuki Hospital, Hachioji, Japan; ^13^ Department of Psychiatry, Hirakawa Hospital, Hachioji, Japan; ^14^ Department of Psychiatry, Juntendo University Nerima Hospital, Nerima-ku, Japan; ^15^ Department of Psychiatry, International University of Health and Welfare Graduate School, Narita, Japan

**Keywords:** bipolar disorder, childbearing-age women, congenital malformations, pharmacoepidemiology, pregnancy, valproate

## Abstract

**Background:**

Accumulating evidence has shown that valproate has the greatest teratogenic potential for increasing the risk of major congenital malformations, such as neural tube defects, cleft palate, and neurodevelopmental disability. Although valproate is a pharmacological option for acute mania and is used as a stabilization drug for patients with bipolar disorder, some global guidelines state that valproate should not be used for girls or women of childbearing age with bipolar disorder. We investigated patterns in psychiatrists' prescription of valproate for bipolar female patients of childbearing age in Japan.

**Methods:**

From March to May 2018, we conducted a questionnaire survey among psychiatrists from all prefectures in Japan on psychiatric practice as it relates to major depression and bipolar disorder throughout women's life. The questionnaire had two parts: (1) assessment of participating psychiatrists' backgrounds and attitudes toward patients and (2) their patterns of prescription of psychotropics for female patients with mood disorders across generations and periods of pregnancy. Each question item had four response options: “not at all,” “rarely,” “sometimes,” and “frequently.” We examined patterns of prescription for childbearing-aged women (late adolescence/young adulthood aged 18–24 years, childbearing-age, older adults aged 25–49 years) and pregnant women.

**Results:**

In total, 571 psychiatrists (427 males, 123 females, and 21 unknowns) responded appropriately to the questionnaire, including 320 who examined at least one or more late adolescence/young adulthood bipolar women. Approximately 70% of psychiatrists answered that they frequently or sometimes prescribed valproate for bipolar women of childbearing age [late adolescence/young adulthood: not at all, *n* = 23 (7.5%); rarely, *n* = 69 (22.5%); sometimes, *n* =116 (37.8%); and frequently, *n* = 99 (32.2%); childbearing-age, older adults: not at all, *n* = 13 (2.7%); rarely, *n* = 67 (13.8%); sometimes, *n* = 185 (38.1%); and frequently, *n* = 220 (45.4%)]. The proportion of general hospital psychiatrists who answered “not at all” or “rarely” to the frequency of their valproate prescriptions was higher than that of psychiatrists working in other medical facilities (*χ* ^2^(3) = 18.2, *p* < 0.001).

**Conclusion:**

Most psychiatrists frequently or sometimes prescribe valproate for women of childbearing age in Japan.

## Introduction

Bipolar disorder frequently emerges in the late teens and young adults (1–3) and its prevalence in males and females is the same (4). The nature, course, and prognosis of bipolar disorder include: (a) a tendency toward remission and recurrent mood episodes (5), (b) frequent comorbidities such as substance use and anxiety disorders (6), (c) decreased quality of life and neurocognitive functioning in various domains such as work and family life (7, 8), and (d) high mortality characterized by suicide (9, 10) and general medical conditions (11). Therefore, the burden of illness is serious in young bipolar patients that need continuous, ongoing management.

Pharmacological treatment plays a crucial role in the continuous management of patients with bipolar disorder. According to several worldwide guidelines (12–14), pharmacology may be in the form of therapies for any mood episodes, including mania and depression, and as continued treatment for the prevention of any episodes in the maintenance phase. However, among girls, women of childbearing age, and pregnant women with bipolar disorder, continuous medication is often difficult to successfully administer; hence, the pharmacological strategy for these patients must be different from that for other patients due to the risks of congenital malformations. Several guidelines for the management of bipolar disorder such as the National Institute for Health and Care Excellence: bipolar disorder: assessment and management [CG185] (NICE), last updated April 2018 (NICE 185 guideline) (15), and the International College of Neuro-Psychopharmacology (CINP) treatment guidelines for Bipolar disorder in adults (CINP-BD-2017) (12) commonly state that valproate should not be used for women of childbearing age due to its teratogenic potential.

Although valproate is one of the pharmacological options for the treatment of acute mania and the stabilization in patients with bipolar disorder, accumulating evidence has shown that valproate, among other pharmacological treatments for bipolar disorder, has the greatest teratogenic potential as it increases the risk of major congenital malformations, such as neural tube defects, including spina bifida {odds ratio [OR] = 12.7 [95% confidence interval (CI), 7.7 to 20.7]}, and cleft palate [OR = 5.2 (95% CI, 2.8 to 9.9)] (16, 17), and neurodevelopmental disability (18, 19). The CINP-BD-2017 states that valproate is not suitable for women of childbearing age. The NICE 185 guideline (15) and the UK National Institute for Health and Care Excellence Clinical Guideline 192, antenatal and postnatal mental health clinical management and service guidance, last updated April 2018 (NICE 192 guideline) (20), also state that unless alternative medications are not suitable, women (including prepubertal young girls) or the women without a pregnancy prevention program certificate should not undergo valproate treatment. Moreover, in November 2014, due to the emerging findings that prenatal exposure to valproate lead to neurodevelopmental adversities such as intellectual disability (21, 22), the European Medicines Agency issued a warning on regulatory restrictions regarding the use of valproate on girls and women of potential childbearing age unless other treatments are ineffective or not tolerated.

Our recent study that investigated the top 11 most frequently prescribed anticonvulsants and lithium using the National Insurance claims database in Japan from April 2014 to March 2015, showed that valproate was the most prescribed major anticonvulsant for female outpatients of childbearing age, and was prescribed slightly lesser compared to same-aged men. However, this data preceded the publication of the above-mentioned guidelines (23). The results of our study raise concerns as to whether most psychiatrists or physicians in Japan properly prescribe valproate for childbearing-aged women, who must be cautioned with regard to its high teratogenic potential; and if they were asked adequate questions regarding their methods of contraception during treatment. Based on the findings of our previous study, we hypothesized that most psychiatrists and physicians do not pay close attention to the possibility of pregnancy, or the method of contraception, when they prescribe valproate for childbearing-aged women. We also hypothesized that psychiatrists who pay attention to patients' reproductive potential in their clinical practice only very cautiously consider prescribing valproate to bipolar women of childbearing age.

The aim of this study was to identify psychiatrists' patterns of prescribing valproate for bipolar female patients of childbearing age (in their late adolescent years and late forties), compared to those who are pregnant, in Japan. We also investigated the relationship between characteristics and reproduction-related interviews in the consultation of psychiatrists and their frequency of valproate prescription. To assess this, this study was performed through a questionnaire survey about psychiatric practices in major depression and bipolar disorders throughout the stages of a woman's life; this was conducted among psychiatrists in all prefectures in Japan from March to May 2018. This study also examined the frequency of prescription of other mood stabilizers [carbamazepine, lamotrigine, and lithium, which has teratogenic effects in humans and potentially causes cardiac malformations, such as Ebstein anomaly (24)], antipsychotics, and antidepressants in bipolar women of childbearing age and in pregnant women; we compared them with prescriptions for valproate.

## Materials and Methods

### Study Design and Participants

A cross-sectional study was conducted among psychiatrists who belong to the following associations: Association of Japan Psychiatric Clinics: Tokyo branch and Chiba branch, Association of Japan Psychiatric Hospitals: Ibaraki branch, Chiba branch, and Tokyo branch, JSGHP; Japanese Society of General Hospital Psychiatry in 2018.

A total of 1414 medical institutions (963 General hospitals, 343 mental clinics, and 108 psychiatric hospitals) were chosen from the list of cooperation associations and 4,816 questionnaires were mailed through the postal service between March and May 2018. There were 571 respondents (427 male, 123 female, and 21 unknowns; mean age = 45.9 years, SD = 10.8). Psychiatrists who did not see patients of childbearing age (18 to 49 years) were excluded. Of 571 respondents, 320 (56.0%) and 497 (87.0%) psychiatrists met the eligible criteria for evaluating the prescription pattern among female bipolar patients in late adolescence/young adulthood (ages 18–24 years) and in early or middle age, respectively. Of 571 respondents, 571 (100.0%) psychiatrists met the eligible criteria for evaluating the prescription pattern of female bipolar patients who were pregnant.

At the beginning of the questionnaire, we declared the objectives of the study and our commitment to confidentiality. Therefore, completing and mailing back the questionnaire were considered to reflect written informed consent regarding participation in this study. The questionnaire survey of this study was performed anonymously. The study's protocol was approved by the ethics committees of the Graduate School of Medicine and School of Medicine, Chiba University (20 November 2017).

### Measures

#### Sociodemographic Characteristics and Prescription Patterns of Participants

The questionnaire was used to assess participating psychiatrists' backgrounds, attitudes toward patients, and prescribing behavior. Regarding psychiatrists' backgrounds, participants were asked about their demographic data, including sex, age, years of clinical experience, hospital facility, and the number of patients with depression and bipolar disorder they treat per month. Regarding attitudes toward patients, the psychiatrists rated how often they focused on problems specific to life stages when counseling their patients. The question was followed by four response options: “not at all,” “rarely,” “sometimes,” and “frequently.” In this study, we focused on measurements of psychiatrists' attitudes toward patients' fertility by asking how often psychiatrists inquire about the following: any menstrual disorder, whether patients wanted to bear children, methods of contraception, and feelings of rejection for medication during pregnancy and breast-feeding periods. In their prescribing behavior, psychiatrists were requested to indicate how often they prescribed 16 types of medicine, depending on the patients' life stages. The life cycle was divided into 7 stages depending on the age: ages 0–11 corresponded to childhood, ages 12–17 to pubescence, ages 18–24 to late adolescence/young adulthood, ages 25–49 to early middle age, ages 50–59 to late middle age, ages 65 and over to late adulthood, and the pregnancy period. The number of the questions was 326 in total, consisting of 35 questions on the psychiatrists' backgrounds including sociodemographic data, 67 on their attitudes toward patients, and 224 on prescribing behavior (**Supplementary Material**). In this study, we focused on childbearing age and the pregnancy period, and defined childbearing age as being from 18 to 49 years and considered it to correspond to late adolescence and early middle age stages.

The 16 types of drugs were of four categories: antidepressants, mood stabilizers, antipsychotics, and Kampo. A full list of the medications is as follows: antidepressants: selective serotonin reuptake inhibitors, serotonin and norepinephrine reuptake inhibitors, mirtazapine, tricyclic antidepressants, tetracyclic antidepressants. Mood stabilizer: lithium carbonate, sodium valproate, carbamazepine and lamotrigine. Antipsychotics: typical antipsychotic, atypical antipsychotic; risperidone, olanzapine, quetiapine, aripiprazole and others atypical antipsychotics. Kampo (herbal medicine).

### Primary and Secondary Outcomes

The primary outcome was to identify the psychiatrists' patterns of prescribing valproate to female bipolar patients of childbearing age, compared to those who are pregnant, and the relationship between the psychiatrists' characteristics and reproduction-related interviews and patterns of valproate prescription. The secondary outcomes were to clarify the frequency of the prescription of other mood stabilizers (especially lithium), antipsychotics, and antidepressants in bipolar women of childbearing-age and in pregnant women.

### Statistical Analysis

We first performed chi-squared (*χ* ^2^) tests for categorized variables to analyze categorical data between the current and previous affiliations of psychiatrists (i.e., general hospitals or other medical facilities) and the frequency of prescription of valproate to female bipolar patients in late adolescence/young adulthood. Categorical data were compared using the χ^2^ tests followed by a residual analysis for multiple comparison (25). Additionally, we calculated the partial correlations, controlling for sex, between years of clinical experience, valproate prescription, and psychiatrists' attitude toward bipolar patients of childbearing-age and pregnant women. For all tests, a two-tailed *p* < 0.05 was considered statistically significant. All analyses were conducted using SPSS 22 (Arbuckle, 2013).

## Results

### Psychiatrists’ Trends for Prescribing Psychotropic Drugs for Female Bipolar Patients of Childbearing Age

A total of 320, 497, and 571 psychiatrists participated in the assessment of prescription trends for late adolescence/young adulthood, early middle age, and pregnant female patients with bipolar disorder, respectively. The participants' demographic characteristics are shown in **Table 1**.

**Table 1:** Social and demographic characteristics of participants (*N* = 571).

	*n*	(%)
Male	427	(74.8)
Female	123	(21.5)
Unknown	21	(3.7)
Age (years)		
Mean	45.9	
Standard deviation	10.8	
Years of clinical experience		
<11 years	181	(33.1)
11 years to under 21	180	(32.9)
21 years to under 31years	111	(20.3)
More than 30 years	61	(13.7)
Unknown	24	(4.2)
Facilities		
general hospital	307	(53.8)
psychiatric hospital	151	(26.4)
clinic	62	(10.9)
others	7	(1.2)
Unknown	21	(3.7)
Specialized clinical department		
Psychiatry (general)	537	(94.0)
Psychosomatic medicine	27	(4.7)
Child psychiatry	33	(5.8)
Others	7	(1.2)

In general, the psychotropic drug we chose was mostly prescribed to female bipolar patients of childbearing-age than to pregnant patients. Among female patients of childbearing age, early middle age patients received more psychotropic drug prescriptions than late adolescence/young adulthood patients, as shown in **Table 2**.

**Table 2:** Row point questionnaire answers of drug prescription for bipolar female patients.

	Childbearing age																Pregnant women (*N* = 571)							
	Late adolescence/Young adulthood (18-24 years; *N* = 320)								Childbearing-age, older adults (25-49 years; *N* = 497)															
	Not at all		Rarely		Sometimes		Frequently		Not at all		Rarely		Sometimes		Frequently		Not at all		Rarely		Sometimes		Frequently	
	*n* (%)								*n* (%)								*n* (%)							
Mood stabilizers																								
Valproate	23	(7.5)	69	(22.5)	116	(37.8)	99	(32.2)	13	(2.7)	67	(13.8)	185	(38.1)	220	(45.4)	387	(75.3)	79	(15.4)	31	(6.0)	16	(3.1)
Lithium carbonate	15	(5.8)	54	(17.5)	125	(40.6)	111	(36.2)	7	(1.4)	42	(8.7)	181	(37.3)	255	(52.6)	382	(74.0)	77	(14.9)	37	(7.2)	20	(3.9)
Carbamazepine	109	(35.9)	102	(33.6)	74	(24.3)	19	(6.3)	122	(25.5)	157	(32.8)	148	(30.9)	52	(11.9)	402	(77.9)	78	(15.2)	30	(5.8)	3	(0.6)
Lamotrigine	32	(10.5)	57	(18.7)	126	(41.3)	90	(29.5)	40	(8.4)	75	(15.7)	209	(43.6)	155	(32.4)	221	(42.9)	119	(23.1)	124	(24.1)	51	(9.9)
Antidepressants																								
SSRIs^a^	136	(44.2)	115	(37.3)	43	(14.0)	14	(4.5)	137	(28.2)	186	(38.3)	124	(25.5)	39	(8.0)	288	(55.7)	167	(32.3)	50	(9.7)	10	(1.9)
SNRIs^b^	108	(35.2)	120	(39.1)	63	(20.5)	16	(5.2)	184	(37.9)	173	(35.6)	96	(19.8)	32	(6.6)	323	(62.5)	152	(29.4)	34	(6.6)	8	(1.5)
Mirtazapine	122	(39.7)	117	(38.1)	55	(17.9)	13	(4.2)	168	(34.8)	167	(34.6)	121	(25.1)	27	(5.6)	324	(62.8)	146	(28.3)	39	(7.6)	7	(1.4)
Tricyclic antidepressants	233	(76.4)	61	(20.0)	10	(3.3)	1	(0.3)	341	(70.6)	106	(21.9)	33	(6.8)	3	(0.6)	455	(88.5)	53	(10.3)	5	(1.0)	1	(0.2)
Tetracyclic antidepressants	216	(70.4)	65	(21.2)	24	(7.8)	2	(0.6)	309	(63.8)	118	(24.4)	51	(10.5)	6	(1.2)	423	(82.1)	74	(14.4)	17	(3.3)	1	(0.1)
Antipsychotics																								
Risperidone	54	(17.7)	102	(33.4)	121	(39.7)	28	(9.2)	64	(13.3)	150	(31.2)	208	(43.2)	59	(12.3)	200	(38.8)	190	(36.8)	106	(20.5)	20	(3.9)
Olanzapine	20	(6.5)	79	(25.7)	144	(46.9)	64	(20.8)	20	(4.2)	93	(19.3)	242	(50.3)	126	(26.2)	161	(31.0)	180	(34.7)	144	(27.7)	33	(6.4)
Quetiapine	17	(5.5)	71	(23.1)	147	(47.9)	71	(23.1)	20	(4.1)	73	(15.1)	250	(51.9)	139	(28.8)	144	(27.7)	165	(31.8)	160	(30.8)	50	(9.6)
Aripiprazole	10	(3.3)	47	(15.6)	164	(53.6)	85	(27.8)	11	(2.3)	54	(11.2)	264	(54.8)	153	(31.7)	110	(21.3)	177	(34.3)	180	(34.9)	49	(9.5)
Other atypical antipsychotics	109	(36.2)	109	(36.2)	66	(21.9)	17	(5.6)	170	(36.1)	150	(31.8)	120	(25.5)	31	(6.6)	286	(56.5)	148	(29.2)	65	(12.8)	7	(1.4)
Typical antipsychotics^c^	171	(57.2)	69	(23.1)	42	(14.0)	17	(5.7)	229	(48.8)	124	(26.4)	85	(18.1)	31	(6.6)	342	(67.2)	114	(22.4)	43	(8.4)	9	(1.8)
Kampo medicine (Herbal medicine)	99	(32.7)	98	(32.3)	79	(26.1)	27	(8.9)	156	(33.0)	134	(28.3)	144	(30.4)	39	(7.8)	211	(41.1)	152	(29.6)	111	(21.6)	39	(7.6)

a SSRIs, selective serotonin reuptake inhibitors (e.g., paroxetine hydrochloride hydrate, sertraline hydrochloride, escitalopram oxalate, and fluvoxamine maleate).

b SNRIs, serotonin noradrenalin reuptake inhibitors (e.g., duloxetine hydrochloride, milnacipran hydrochloride, and venlafaxine hydrochloride).

c Typical antipsychotics (e.g., Chlorpromazine hydrochloride, haloperidol, levomepromazine maleate, sultopride hydrochloride, timiperone, and zotepine).

In the questionnaire, the psychiatrists' answers regarding the prescription of valproate to late adolescence/young adulthood female patients with bipolar disorder were the following: 23 psychiatrists responded not at all, *n* = 23 (7.5%); rarely, *n* = 69 (22.5%); sometimes, *n* =116 (37.8%); and frequently, *n* = 99 (32.2%). Regarding the prescription of valproate to childbearing-aged, older female patients with bipolar disorder the answers were the following: 13 psychiatrists responded not at all, *n* = 13 (2.7%); rarely, *n* = 67 (13.8%); sometimes, *n* = 185 (38.1%); and frequently, *n* = 220 (45.4%). On avoiding prescription of valproate to pregnant patients: 387 psychiatrists responded “not at all” (75.3%) while 126 responded “rarely”, “sometimes”, and “frequently” (24.7%).

Regarding the prescription of lithium to late adolescence/young adulthood patients with bipolar disorder, 15 psychiatrists responded not at all, *n* = 15 (5.8%); rarely, *n* = 54 (17.5%); sometimes, *n* =125 (40.6%); and frequently, *n* = 111 (36.2%). Regarding the prescription of lithium for childbearing-aged, older female patients; 7 psychiatrists responded not at all, *n* = 7 (1.4%); rarely, *n* = 42 (8.7%); sometimes, *n* = 181 (37.3%); and frequently, *n* = 255 (52.6%). Regarding lithium prescription to pregnant patients: 382 psychiatrists responded “not at all” (74.0%), while 134 responded “rarely,” “sometimes,” and “frequently” (26.0%).

In addition, regarding the prescription patterns of other medications, including other mood stabilizers (lamotrigine, carbamazepine) and antipsychotics for pregnant women, more than half (55.6%) the psychiatrists responded, “not at all” or “rarely” (**Table 2**).

### Affiliation of Psychiatrists and Prescription of Valproate for Female Bipolar Patients in Late Adolescence/Young Adulthood

The *χ* ^2^-test showed that there were significant differences in the current affiliation of psychiatrists and valproate prescriptions to female bipolar patients in late adolescence/young adulthood, as shown in **Table 3** [*χ* ^2^(3) = 18.2, *p* < 0.001]. The Haberman-type residual analysis showed that psychiatrists working for general hospitals reported that they prescribed valproate less frequently (with either “Not at all” or “Rarely” in the questionnaire) than that of psychiatrists working for other medical facilities and hospitals. Additionally, the psychiatrists working for general hospitals answered “frequently,” regarding valproate prescription, were associated with significantly negative standardized residuals, which was less than psychiatrists working for other medical facilities or hospitals. However, in their previous affiliations, there were no differences between general hospitals and other medical facilities in terms of valproate prescriptions.

**Table 3:** Affiliation of psychiatrist and valproate prescription for female bipolar patients in late adolescence/young adulthood (18-24 years).

	Prescription of Valproate								*χ* ^2^	*p*
	Not at all		Rarely		Sometimes		Frequently			
Current Affiliation									**18.2**	**0.001**
General Hospitals										
Oserved [*n* (Row %)]	20	(11.3)	49	(27.7)	57	(32.2)	51	(28.8)		
Expected	14.3		37.5		65.1		60.1			
Adjusted standardized residual	**2.3**		**3.2**		−1.9		**−2.2**			
Other medical facilities										
Oserved [*n* (Row %)]	6	(4.2)	19	(13.2)	61	(42.4)	58	(40.3)		
Expected	11.7		30.5		52.9		48.9			
Adjusted standardized residual	−2.3		−3.2		1.9		2.2			
Previous Affiliation									5.3	0.2
General Hospitals										
Oserved [*n* (Row %)]	24	(8.1)	67	(22.7)	106	(35.9)	98	(33.2)		
Expected	23.9		63		108		100.2			
Adjusted standardized residual	0.1		2.3		−1.0		−0.9			
Other medical facilities										
Oserved [*n* (Row %)]	2	(7.7)	1	(3.8)	12	(46.2)	11	(42.3)		
Expected	2.1		5.5		9.6		8.8			
Adjusted standardized residual	−0.1		−2.3		1.0		0.9			

p value was calculated by the Chi-square (χ^2^) tests. Bold numbers are statistically significant.

### Partial Correlation Between Valproate Prescribing Action and Psychiatrists’ Backgrounds

We calculated the partial correlations, controlling for sex, between years of clinical experience, valproate prescription, and psychiatrists' attitude toward bipolar patients of childbearing age and in pregnancy (**Table 4**). The psychiatrists' years of experience was found to be positively and significantly correlated with a valproate prescription in late adolescence/young adulthood (18-24 years) (*r* = 0.13, *p* < 0.05). Conversely, in childbearing-aged, older adults (25–49 years) and pregnant women, there is no relationship between the two variables. There is no relationship between the contents of reproduction-related medical interviews and valproate prescriptions in childbearing age or pregnancy. In addition, there were no relationships between reproduction-related medical interviews and prescriptions of other psychotropics in childbearing age or pregnancy.

**Table 4:** Partial correlation between valproate prescription and psychiatrists' attitude toward bipolar patients in childbearing age and pregnancy.

	Prescription of Valproate		
	Late adolescence/Young adulthood (18–24 years)	Childbearing-age, older adults (25–49 years)	Pregnant women	
Years of clinical experience	.13^*^	−.01	.01
Reproductive-related medical interview			
Abnormal menstruation	−.02	−.03	−.04
Having desire to bear children	−.03	.03	−.04
Method of contraception	.02	.01	.08
Internal medicine avoiding during pregnancy and breast feeding	.09	.06	−.05

Partial Correlation coefficients were controlled for sex. ^*^p < .05. Bold numbers are statistically significant.

## Discussion

In this study, three important findings about psychiatrists' prescription patterns of valproate for female bipolar patients of childbearing age in Japan deserve to be mentioned. First, this study demonstrates that 70% of psychiatrists responding to the questionnaire sheet answered that they frequently or occasionally prescribed valproate for women of childbearing age in Japan, although most answered that they did not prescribe for women who were pregnant. Specifically, 32% of psychiatrists answered that they frequently prescribed valproate for women aged 18 to 24 years, and 38% occasionally prescribed it for same-aged women. Thirty percent of psychiatrists answered they rarely prescribed valproate, or that they did not at all prescribe it. Second, psychiatrists affiliated with general hospitals answered that they tended to refrain from prescribing valproate for childbearing-aged women compared to those affiliated with other medical facilities such as psychiatric hospitals or private clinics. Third, the frequencies of reproduction-related medical interviews were not correlated with the tendency to prescribe valproate to women of childbearing age, although years of psychiatric experience was positively correlated with this.

This questionnaire study conducted from March to May 2018 reveals the possibility that more than half of psychiatrists frequently or occasionally prescribed valproate for women of childbearing age in Japan. This finding is consistent with our previous study investigating the frequently prescribed tablets for childbearing-aged outpatients in Japan (23). On April 6, 2017, in reference to pharmacological treatments of women in the perinatal period, the Japanese Society of Perinatal Mental Health published the Perinatal Mental Health Consensus Guide 2017 on the website (http://pmhguideline.com/, in Japanese only); stating that valproate should not be prescribed to women of childbearing age. However, the society is small due to the small number of psychiatrists. Additionally, there were only 20 regular members at that time, and midwives constituted a large proportion of the regular members of the society (over 100 people). In other countries, such as Finland (26), Ireland (27), and Germany (28), valproate prescription has slightly declined, although the data from these studies are not limited to pharmacological treatments of bipolar disorder. In the UK, at the same time as the last updated NICE 192 guideline, the Medicines and Healthcare Products Regulatory Agency in April 2018, stated that valproate use would be rigidly regulated and that valproate treatment must not be used for women, including prepubertal young girls, unless alternative medications are not suitable or when the women are part of a pregnancy prevention program (29). Considering that fetal exposure to valproate in the first trimester must be avoided and that approximately 40% of pregnancies are unplanned or unintended (30), psychiatrists and physicians should not, in principle, prescribe valproate to girls and childbearing-aged women with bipolar disorder. Furthermore, since changes in valproate prescription patterns among these countries may be slow, societies, associations, and related guidelines that directly influence psychiatrists or physicians are expected to clearly share warnings about the dangers of valproate prescriptions to girls and childbearing-aged women with bipolar disorder in Japan and other countries. Additionally, the Japanese authorized societies or licensing agencies should issue warnings about teratogenic risks of valproate prescription not only for pregnant women but also childbearing-aged women.

In this study, psychiatrists working at general hospitals answered that they refrained from prescribing valproate to childbearing-aged women compared to those affiliated with other medical facilities, such as psychiatric hospitals and private clinics. There are two main possible interpretations of this result. Freudenreich and Kontos report that consultation-liaison psychiatrists have better opportunities and situations for learning other medical specialties through their interdisciplinary collaborative work (31). This may make sense because psychiatrists working at general hospitals, compared to other institutions, have better chances and experience in consulting for pregnant women with psychiatric diseases at their hospitals, which either have a birth center or obstetricians working in them. There are other reasons why psychiatric diseases and symptomatic and social severities of patients are different across general hospitals and other institutes. For instance, in/outpatients being treated by psychiatrists in psychiatric hospitals may have more severe symptomatology, impaired social ability due to long-term hospitalization, and compromised cognitive performance, including intellectual disability, than those in general hospitals. Therefore, it is difficult for such patients to become pregnant; hence, psychiatrists prescribe valproate to them. However, in the real-world, women with severe mental illness such as schizophrenia and autism are fertile, do get pregnant, and have newborns (32). Therefore, the societies and associations that largely influence psychiatrists, regardless of the type of medical institute, should provide warnings about valproate prescription for girls and women of childbearing age.

In addition, the frequencies of reproduction-related medical interviews were not correlated with the tendency to prescribe valproate to women of childbearing age, although years of psychiatric experience was positively correlated with this. These results were unexpected because our hypothesis was that psychiatrists who usually paid attention to future conceptions in their consultations of childbearing-age women would often not have prescribed valproate to these women. The interpretation of this result was that there may be two groups of psychiatrists regarding pregnancy; (1) those who refrained from prescribing valproate to childbearing-aged women, and (2) the other ones who prescribed it with caution. It is difficult to interpret the positive correlation between years of psychiatric experience and the tendency to prescribe valproate to childbearing-age women. This result is limited because of the lack of surveillance about real-world childbearing-aged women prescription of valproate, and the lack of assessment of each psychiatrist's valproate prescription dosage. Therefore, senior psychiatrists with enough clinical experience may have the opportunity to examine childbearing-aged women with more refractory bipolar disorder than junior ones. Further studies are needed to clarify this.

Regarding other psychotropics, especially lithium and carbamazepine prescriptions, the NICE 192 guideline cautions against use on girls and childbearing-aged women (20); however, the frequencies of lithium prescriptions were similar to those of valproate. Approximately 77% of responding psychiatrists answered that they frequently or sometimes prescribed lithium to bipolar women of childbearing age in Japan, and carbamazepine was not as frequently prescribed. These findings are consistent with our previous study (23). Fetal exposure to lithium in the first trimester is associated with increased risk of cardiac malformations (24). According to the NICE guideline, unless lithium is recommended to women planning pregnancies who do not clinically respond to other antipsychotics or mood stabilizers, but do respond to lithium, it should be avoided in the first trimester (20). Consequently, although the adverse effects of lithium use on the fetus are known to be dose-dependent, the present finding demonstrates that many psychiatrists tend to easily prescribe lithium to childbearing-aged women with bipolar disorder in Japan. Therefore, psychiatrists should be more careful when continuously prescribing lithium to girls and women of childbearing age.

Additionally, the present study shows that most psychiatrists are rather reluctant to prescribe medications including lamotrigine and some atypical antipsychotics with efficacy and/or relative safety in the treatment and prevention of mood episodes in pregnant women with bipolar disorder in Japan. Among major mental disorders, bipolar disorder is the highest risk group with regard to relapse and readmission during the postpartum period (33). Wesseloo et al.'s meta-analysis demonstrates that the postpartum relapse rates of bipolar women who were medication free during pregnancy (medication free during pregnancy: 66%, 95% CI, 57 to 75) were significantly higher than that of those who used prophylactic medication (using prophylactic medication: 23%, 95% CI, 14 to 37) (34). Considering that the psychiatrists who sometimes or frequently prescribe medications for bipolar female patients during the pregnancy were a minority, the present findings show the existence of evidence-practice gap in pharmacological treatment of pregnant women with bipolar disorder. Psychiatrists should provide more information on the potential risks and benefits of medication treatments of bipolar disorder, and implement a shared decision-making process with bipolar women of childbearing age before pregnancy.

We acknowledge that there are several limitations to this study. First, we were unable to measure the real-world valproate prescriptions (including the dosage) of the responding psychiatrists. Given that some female patients with bipolar disorder who consulted their responding psychiatrist were expected to have various comorbidities, intellectual disabilities, and complications (including epilepsy, early menopause, and bilateral ovarian ablation), valproate prescription may be adequate for them if prescribed with caution. Second, the response rate of 11.8% was low, although 571 psychiatrists responded to this questionnaire in Japan. The biggest possible reason for the low response rate in this survey was the large amount of questions in the questionnaire (326 items in total) since it was developed to investigate overall psychiatric practices in major depression and bipolar disorder throughout the stages of the women's lives. Hence, it was likely that lots of questions caused difficulties for almost 90% candidates to complete the questionnaire sheet. Third, the items about reproduction-related medical interviews were relatively scarce in the questionnaire; hence, this may have influenced the correlations between the answers and frequencies of valproate prescriptions.

## Conclusion

This study demonstrates that most psychiatrists frequently or occasionally prescribed valproate for women of childbearing age in Japan, although almost all answered they did not prescribe valproate to pregnant women. Specifically, 70% of psychiatrists answered that they frequently or sometimes prescribed valproate for women aged 18 to 24 years. Considering that fetal exposure to valproate in the first trimester must be avoided, and that many pregnancies are unplanned or unintended, psychiatrists should not prescribe valproate to girls and women of childbearing age with bipolar disorder.

## Data Availability Statement

The datasets analyzed in this article will be made available by the authors, and to any qualified researcher without undue reservation. Requests to access the datasets should be directed to TH, t.hashimoto1109@gmail.com.

## Ethics Statement

The studies involving human participants were reviewed and approved by the ethics committees of the Graduate School of Medicine and School of Medicine, Chiba University. The patients/participants provided their written informed consent to participate in this study.

## Author Contributions

TH, MamiT, YaS, TakaT, TakeT, HW, MN, and MI contributed substantially to the conception and design of this study. MasuT, TH, and MamiT contributed to organizing the data, formal analysis, writing an original draft. AK, TakeT, KH, and MI contributed to the management of this study as supervisors. SK, AK, SE, YuS, TN, JH, and KH contributed substantially to facilitating data curation and study procedure as investigators. All authors contributed to the interpretation of data, revision of the manuscript, and approved of the submitted manuscript.

## Funding

This work was supported by the Japan Society for the Promotion of Science Grant-in-Aid for Scientific Research C Grant Number 17K10265. The members of this grant were TH, HW, MN, MI, YS, TakaT, and TakeT.

## Conflict of Interest

TH reported personal fees from research support of a clinical trial that the signant health (former, blacket global) company manages. TakaT has received personal fees from Otsuka, GlaxoSmithKline, MSD, Dainippon-Sumitomo, Janssen, Eisai, Eli Lilly, Daiichi-Sankyo, Shionogi, and Tanabe-Mitsubishi. KH has received lecture honoraria for Dainippon-Sumitomo, Janssen, Meiji Seika, MSD, Otsuka, Takeda, and Tanabe-Mitsubishi, and has served as a consultant for Dainippon-Sumitomo, MSD, and Meiji Seika. HW has received speaker's honoraria for MSD and Eli Lilly. TakeT has received honoraria from Otsuka Pharmaceutical Co., Ltd. and Mochida Pharmaceutical Co., Ltd as potentially influencing but not directly related to this work. MI received consultant fees from Janssen, Elilly, Otsuka, and Meiji Seika Pharma and reports honoraria from Janssen, Eli Lilly, Otsuka, Meiji Seika Pharma, Astellas, Dainippon Sumitomo, Ono, Mochida, MSD, Eisai, Daiichi-Sankyo, Novartis, Teijin, Shionogi, Hisamitsu, and Asahi Kasei.

The remaining authors declare that the research was conducted in the absence of any commercial or financial relationships that could be construed as a potential conflict of interest.
