# Supplementary material for: Patterns in Psychiatrists’ Prescription of Valproate for Female Patients of Childbearing Age With Bipolar Disorder in Japan: A Questionnaire Survey
Source: Front Psychiatry. 2020 Apr 15;11:250. doi: 10.3389/fpsyt.2020.00250 (PMC7176044; doi:10.3389/fpsyt.2020.00250)
Supplement: Supplementary file 1 [file Presentation_1.pdf]

For psychiatrists nationwide

A survey of depression and bipolar disorder treatment based on women's life stages.

- Diagnosis and treatment continue to change as awareness of depression and bipolar disorder spreads. Although there are a wide range of topics relating to the two aforementioned disorders, the present questionnaire is conducted with the purpose of identifying current treatment against the background of women's life stages notably, childhood /pubescence, late adolescence/young adulthood, pregnancy/childbirth and child raising, menopause, and the older generation.
- Survey results will be de-identified, aggregated and analyzed, with results presented at academic events and complied in an academic research paper. Individual circumstances will not be published.
- We would like to ask for your cooperation in order to investigate and survey current treatments for depression and bipolar disorder against a background of women's life stages.
- Please decide whether or not to cooperate with the survey, based on your own free will. Non-participation will entail no disadvantage. If you consent to participate in this survey, please return the completed questionnaire by post in envelope provided. In the event of withdrawal of consent after the return of the questionnaire paper, data cannot be deleted, as the anonymous nature of the survey will make identification of the actual answer sheet difficult. Your understanding in this matter is appreciated.
- When filling out your answers please circle the correct number or write the correct number in the brackets(　).

[Survey Outline]

○ Aim: To identify current treatment for depression and bipolar disorder based on the life stages of female patients.

○ Participants: Psychiatrists nationwide

○ An anonymous survey was sent to psychiatrists nationwide, with the responses returned by post.

(For all Inquiries)　Phone: +81-43-226-2148, Fax: +81-43-226-2150 (direct), Email: wlifestage@gmail.com

A) Please answer these questions about yourself

① Specialist Department (Multiple answers possible)

(1)Psychiatry (General Adult) (2) Psychosomatic medicine 3. Child psychiatric (4) Other

(2) Age ( )years-old

(3) Gender 1.Male 2. Female

(4) Years since obtaining a doctor's license (years)

(5) Years of psychiatric clinical experience (years)

＊ Doctors who gained their doctors license after 2004 (Undertook super rotation training)are not include their early training period.

(6) Please circle all the types of medical institutions you have had the experience of working in full-time.

1. General Hospital 2. Psychiatric Hospital 3. Clinic 4. Other ( )

(7) Current affiliation

1. General Hospital 2. Psychiatric Hospital 3. Clinic 4. Other ( )

(8) Days per week worked at current workplace ( )days/week

(9) Prefecture of current workplace( )

10. a) Number of depression patients currently examined (person/month).

(Please circle one response from 1～5)

1. ～ 25 people 2. ～50 people 3. ～75 people 4. ～ 100 people 5. More than 100 people

b) Number of depression patients by gender and age

(Please circle one answer 1 ～ 4 for each age bracket)

[Male depression patients] (people/month).

0～1 1 years	1． 0 people	2． 1～9 people	3． 1 0～1 9 people	4． More than 19
1 2～1 7 years	1． 0 people	2． 1～9 people	3． 1 0～1 9 people	4． More than 19
1 8～2 4 years	1． 0 people	2． 1～9 people	3． 1 0～1 9 people	4． More than 19
2 5～4 9 years	1． 0 people	2． 1～9 people	3． 1 0～1 9 people	4． More than 19
5 0～6 4 years	1． 0 people	2． 1～9 people	3． 1 0～1 9 people	4． More than 19

Over６５ years	１． 0 people	２． 1～9 people	３． １０～１９people	４． More than 19

【Female depression patients】(people/month)

0～11 years	１． 0 people	２． 1～9 people	３． １０～１９people	４． More than 19
12～17 years	１． 0 people	２． 1～9 people	３． １０～１９people	４． More than 19
18～24 years	１． 0 people	２． 1～9 people	３． １０～１９people	４． More than 19
25～49 years	１． 0 people	２． 1～9 people	３． １０～１９people	４． More than 19
50～64 years	１． 0 people	２． 1～9 people	３． １０～１９people	４． More than 19
Over 65 years	１． 0 people	２． 1～9 people	３． １０～１９people	４． More than 19

⑪ a) Number of bipolar disorder patients currently examined (person/month).

(Please circle one response from 1～5)

1. ～25 people　2． ～50 people　3. ～75 people　4. ～100 people　5. More than 100 people

b) Number of bipolar disorder patients by gender and age

(Please circle one answer 1 ～ 4 for each age bracket)

[Male bipolar disorder patients] (people/month).

０～１１years	１． 0 people	２． １～９people	３． １０～１９people	４． More than 19
１２～１７ years	１． 0 people	２． １～９people	３． １０～１９people	４． More than 19
１８～２４ years	１． 0 people	２． １～９people	３． １０～１９people	４． More than 19
２５～４９ years	１． 0 people	２． １～９people	３． １０～１９people	４． More than 19
５０～６４ years	１． 0 people	２． １～９people	３． １０～１９people	４． More than 19
６５years 以上	１． 0 people	２． １～９people	３． １０～１９people	４． More than 19

【Female bipolar disorder patients】(people/month)

０～１１years	１． 0 people	２． １～９people	３． １０～１９people	４． More than 19
１２～１７ years	１． 0 people	２． １～９people	３． １０～１９people	４． More than 19
１８～２４ years	１． 0 people	２． １～９people	３． １０～１９people	４． More than 19

２５～４９ years	1． 0 people	2． 1～9 people	3． １０～１９people	4． More than 19
５０～６４ years	1． 0 people	2． 1～9 people	3． １０～１９people	4． More than 19
６５years 以上	1． 0 people	2． 1～9 people	3． １０～１９people	4． More than 19

B−１）In day-to-day consultations, which of the following items are noted during the first visit of female depression and bipolar disorder patients and their families? Please circle the most appropriate number.

	**Patient Background**<br>**(<u>First visit</u> checklist for all age groups)**	Do not check	Rarely check	Sometimes check	Do check
1	Family history of mood disorders	1	2	3	4
2	Quality of family relations	1	2	3	4
3	Comorbid physical illness	1	2	3	4
4	Whether they exercise regularly	1	2	3	4
5	History of self-harm behavior	1	2	3	4
6	History of overdosing	1	2	3	4
7	Presence of suicidal thoughts to date	1	2	3	4
8	Abnormalities from prenatal stage ~birth（ neonatal asphyxia, premature birth, cesarean section etc.）	1	2	3	4
9	Early childhood neurodevelopment (０～６years)	1	2	3	4
10	Adjustment to group-based childcare（Kindergarten/Daycare)	1	2	3	4
11	Therapy during the preschool period（Speech Therapist etc.）	1	2	3	4
12	Experience of child abuse	1	2	3	4
13	School avoidance issues	1	2	3	4
14	Bullying issues	1	2	3	4

B-２）In day-to-day consultations, which of the following items are checked during continuing treatment consultations of female depression and bipolar disorder patients and their families? Please circle the most appropriate number.

	**Continuing treatment checklist: Pubescence （１２～１７year-old female patients）**	Do not check	Rarely check	Sometimes check	Do check
1	Menstrual irregularities （menstrual cycle / premenstrual syndrome etc.）	1	2	3	4
2	Anorexic/Bulimic Episodes（Including eating problems）	1	2	3	4
3	What the patient thinks the cause of the problem is.	1	2	3	4
4	Whether the patient has tried discussing the issue with someone	1	2	3	4
5	The patient’s coping strategies	1	2	3	4
6	Occupation choice and concerns(Regular employment, part-time jobs etc.)	1	2	3	4
7	Recent suicidal ideation	1	2	3	4

	**Continuing treatment checklist: Late adolescence/Early adulthood, Early middle age, Middle age （１８～６４year-old female patients）**	Do not check	Rarely check	Sometimes check	Do check
1	Menstrual irregularities （menstrual cycle / premenstrual syndrome /menopause related problems etc.）	1	2	3	4
2	Anorexic/Bulimic Episodes	1	2	3	4
3	What the patient thinks the cause of the problem is	1	2	3	4
4	Whether the patient has tried discussing the issue with someone	1	2	3	4
5	The patient’s coping strategies	1	2	3	4
6	Occupation choice and occupational concerns (Regular employment, part-time jobs etc.)	1	2	3	4

7	Recent suicidal ideation	1	2	3	4
8	For those of child-bearing age, desire to have children	1	2	3	4

	**Continuing treatment checklist:**<br>**Late adolescence/Early adulthood, Early middle age, Middle age**<br>**（１８～６４year-old female patients）**	Do not check	Rarely check	Sometimes check	Do check
9	For those not desiring to have children, type of contraceptive method employed	1	2	3	4
10	Pregnant and breastfeeding patients' reticence toward drug-based treatment	1	2	3	4
11	How much of the household chores the patient is capable of	1	2	3	4
12	If the patient has children (Baby – Elementary school aged), child-rearing support system	1	2	3	4
13	If the patient has children (Baby – Elementary school aged), feelings of love for the child/ren	1	2	3	4
14	If the patient has children (Baby – Elementary school aged), feelings of anger toward the child/ren	1	2	3	4
15	If the patient has children (Baby – Elementary school aged), feelings of rejection toward the child/ren e.g. -"If only this child was not here"	1	2	3	4
16	If the patient has children (Baby – High school aged), instances of child abuse	1	2	3	4
17	Does the patient undergo screening for uterine cancer/breast cancer?	1	2	3	4
18	Menopause symptoms, such as sweating, hot flashes and night sweats	1	2	3	4
19	Discord regarding physical and mental decline	1	2	3	4
20	Care-giver burden	1	2	3	4
21	Abuse toward the person being cared for	1	2	3	4

	Continuing treatment checklist:<br>Late adulthood<br>(Female patients over 65 years-old)	Do not check	Rarely check	Sometimes check	Do check
1	Menstrual irregularities（menopausal issues, menopausal disorders etc.）	1	2	3	4
2	Anorexic/Bulimic Episodes	1	2	3	4
3	What the patient thinks the cause of the problem is	1	2	3	4
4	Whether the patient has tried discussing the issue with someone	1	2	3	4
5	The patient's coping strategies	1	2	3	4
6	Occupation choice and occupational concerns (Regular employment, part-time jobs etc.)	1	2	3	4
7	Recent suicidal ideation	1	2	3	4
8	Do you have something which gives your life a sense of purpose?	1	2	3	4
9	Do you feel satisfied with your life so far?	1	2	3	4
10	Do you have someone to rely on when you are unwell?	1	2	3	4
11	Concerns about decreasing income and assets	1	2	3	4
12	For family members of dementia patients, are they aware of Community-based Integrated Care Centers?	1	2	3	4
13	Are they using the Long-term care insurance system? Have they received Certification for Long-term Care Need?	1	2	3	4
14	Level of independence with activities of daily living	1	2	3	4
15	Do you have any health concerns?	1	2	3	4
16	Do you feel lonely?	1	2	3	4
17	Dissatisfaction with support from family	1	2	3	4

C−１）This question asks about medication prescribed for female depression patients in day-to-day consultations. How much of each medication is prescribed at each female patient's life stage / during pregnancy? Please circle the most appropriate number.

<Female depression patients>

Life stage	Childhood				Pubescence				Alte adolescence /Young adulthood				Early middle age				Middle age				Late adulthood				**Pregnancy**			
Patient age (years)	**0-11**				**12-17**				**18-24**				**25-49**				**50-64**				**65** or more							
Prescribing frequency	Not at all	Rarely	Sometimes	Frequently	Not at all	Rarely	Sometimes	Frequently	Not at all	Rarely	Sometimes	Frequently	Not at all	Rarely	Sometimes	Frequently	Not at all	Rarely	Sometimes	Frequently	Not at all	Rarely	Sometimes	Frequently	Not at all	Rarely	Sometimes	Frequently
＜Anti-depressant medication＞																												
SSRI	1	2	3	4	1	2	3	4	1	2	3	4	1	2	3	4	1	2	3	4	1	2	3	4	1	2	3	4
SNRI	1	2	3	4	1	2	3	4	1	2	3	4	1	2	3	4	1	2	3	4	1	2	3	4	1	2	3	4
Mirtazapine	1	2	3	4	1	2	3	4	1	2	3	4	1	2	3	4	1	2	3	4	1	2	3	4	1	2	3	4
Tricyclic antidepressants	1	2	3	4	1	2	3	4	1	2	3	4	1	2	3	4	1	2	3	4	1	2	3	4	1	2	3	4
Tetracyclic antidepressants	1	2	3	4	1	2	3	4	1	2	3	4	1	2	3	4	1	2	3	4	1	2	3	4	1	2	3	4
＜Mood stabilizer＞																												
Lithium carbonate	1	2	3	4	1	2	3	4	1	2	3	4	1	2	3	4	1	2	3	4	1	2	3	4	1	2	3	4
Sodium Valproate	1	2	3	4	1	2	3	4	1	2	3	4	1	2	3	4	1	2	3	4	1	2	3	4	1	2	3	4
Carbamazepine	1	2	3	4	1	2	3	4	1	2	3	4	1	2	3	4	1	2	3	4	1	2	3	4	1	2	3	4
Lamotrigine	1	2	3	4	1	2	3	4	1	2	3	4	1	2	3	4	1	2	3	4	1	2	3	4	1	2	3	4
＜Antipsychotics＞																												
Typical antipsychotics	1	2	3	4	1	2	3	4	1	2	3	4	1	2	3	4	1	2	3	4	1	2	3	4	1	2	3	4
Atypical antipsychotic																												
Risperidone	1	2	3	4	1	2	3	4	1	2	3	4	1	2	3	4	1	2	3	4	1	2	3	4	1	2	3	4
Olanzapine	1	2	3	4	1	2	3	4	1	2	3	4	1	2	3	4	1	2	3	4	1	2	3	4	1	2	3	4
Quetiapine	1	2	3	4	1	2	3	4	1	2	3	4	1	2	3	4	1	2	3	4	1	2	3	4	1	2	3	4
Aripiprazole	1	2	3	4	1	2	3	4	1	2	3	4	1	2	3	4	1	2	3	4	1	2	3	4	1	2	3	4
others	1	2	3	4	1	2	3	4	1	2	3	4	1	2	3	4	1	2	3	4	1	2	3	4	1	2	3	4
Kanpo	1	2	3	4	1	2	3	4	1	2	3	4	1	2	3	4	1	2	3	4	1	2	3	4	1	2	3	4

Notes:

SSRIs (Selective Serotonin Reuptake Inhibitors) include Paroxetine, Sertraline, Escitalopram, Fluvoxamine.

SNRIs (Serotonin and Norepinephrine Reuptake Inhibitors) include Duloxetine, Milnacipran and Venlafaxine.

Typical antipsychotics include Chlorpromazine, Haloperidol, Levomepromazine, sultopride, timiperone and Zotepine.

C－2）This question asks about medications prescribed for female bipolar disorder patients in day-to-day consultations. How much of each medication is prescribed at each female patient's life stage / during pregnancy? Please circle the most appropriate number. <Female Bipolar Disorder Patients>

Life stage	Childhood				Pubescence				Alte adolescence /Young adulthood				Early middle age				Middle age				Late adulthood				**Pregnancy**			
Patient age (years)	**0-11**				**12-17**				**18-24**				**25-49**				**50-64**				**65** or more							
Prescribing frequency	Not at all	Rarely	Sometimes	Frequently	Not at all	Rarely	Sometimes	Frequently	Not at all	Rarely	Sometimes	Frequently	Not at all	Rarely	Sometimes	Frequently	Not at all	Rarely	Sometimes	Frequently	Not at all	Rarely	Sometimes	Frequently	Not at all	Rarely	Sometimes	Frequently
＜Anti-depressant medication＞																												
SSRI	1	2	3	4	1	2	3	4	1	2	3	4	1	2	3	4	1	2	3	4	1	2	3	4	1	2	3	4
SNRI	1	2	3	4	1	2	3	4	1	2	3	4	1	2	3	4	1	2	3	4	1	2	3	4	1	2	3	4
Mirtazapine	1	2	3	4	1	2	3	4	1	2	3	4	1	2	3	4	1	2	3	4	1	2	3	4	1	2	3	4
Tricyclic antidepressants	1	2	3	4	1	2	3	4	1	2	3	4	1	2	3	4	1	2	3	4	1	2	3	4	1	2	3	4
Tetracyclic antidepressants	1	2	3	4	1	2	3	4	1	2	3	4	1	2	3	4	1	2	3	4	1	2	3	4	1	2	3	4
＜Mood stabilizer＞																												
Lithium carbonate	1	2	3	4	1	2	3	4	1	2	3	4	1	2	3	4	1	2	3	4	1	2	3	4	1	2	3	4
Sodium Valproate	1	2	3	4	1	2	3	4	1	2	3	4	1	2	3	4	1	2	3	4	1	2	3	4	1	2	3	4
Carbamazepine	1	2	3	4	1	2	3	4	1	2	3	4	1	2	3	4	1	2	3	4	1	2	3	4	1	2	3	4
Lamotrigine	1	2	3	4	1	2	3	4	1	2	3	4	1	2	3	4	1	2	3	4	1	2	3	4	1	2	3	4
＜Antipsychotics＞																												
Typical antipsychotics	1	2	3	4	1	2	3	4	1	2	3	4	1	2	3	4	1	2	3	4	1	2	3	4	1	2	3	4
Atypical antipsychotic																												
Risperidone	1	2	3	4	1	2	3	4	1	2	3	4	1	2	3	4	1	2	3	4	1	2	3	4	1	2	3	4
Olanzapine	1	2	3	4	1	2	3	4	1	2	3	4	1	2	3	4	1	2	3	4	1	2	3	4	1	2	3	4
Quetiapine	1	2	3	4	1	2	3	4	1	2	3	4	1	2	3	4	1	2	3	4	1	2	3	4	1	2	3	4
Aripiprazole	1	2	3	4	1	2	3	4	1	2	3	4	1	2	3	4	1	2	3	4	1	2	3	4	1	2	3	4
others	1	2	3	4	1	2	3	4	1	2	3	4	1	2	3	4	1	2	3	4	1	2	3	4	1	2	3	4
Kanpo	1	2	3	4	1	2	3	4	1	2	3	4	1	2	3	4	1	2	3	4	1	2	3	4	1	2	3	4

Notes:

SSRIs (Selective Serotonin Reuptake Inhibitors) include Paroxetine, Sertraline, Escitalopram, Fluvoxamine.

SNRIs (Serotonin and Norepinephrine Reuptake Inhibitors) include Duloxetine, Milnacipran and Venlafaxine.

Typical antipsychotics include Chlorpromazine, Haloperidol, Levomepromazine, sultopride, timiperone and Zotepine.
